# Detection and characterization of copy-number variants from exome sequencing in the DDD study

**DOI:** 10.1016/j.gimo.2024.101818

**Published:** 2024-01-28

**Authors:** Petr Danecek, Eugene J. Gardner, Tomas W. Fitzgerald, Giuseppe Gallone, Joanna Kaplanis, Ruth Y. Eberhardt, Caroline F. Wright, Helen V. Firth, Matthew E. Hurles

**Affiliations:** 1 Wellcome Sanger Institute, Wellcome Genome Campus, Hinxton, United Kingdom; 2 Department of Clinical and Biomedical Sciences, University of Exeter Medical School, Royal Devon and Exeter Hospital, Exeter, United Kingdom; 3 Department of Clinical Genetics, Cambridge University Hospitals NHS Foundation Trust, Cambridge, United Kingdom

**Keywords:** Chromosomal microarrays, Copy-number variation, Exome sequencing, Neurodovelopmental disease, Rare disease

## Abstract

**Purpose:**

Structural variants such as multiexon deletions and duplications are an important cause of disease but are often overlooked in standard exome/genome sequencing analysis. We aimed to evaluate the detection of copy-number variants (CNVs) from exome sequencing (ES) in comparison with genome-wide low-resolution and exon-resolution chromosomal microarrays (CMAs) and to characterize the properties of de novo CNVs in a large clinical cohort.

**Methods:**

We performed CNV detection using ES of 9859 parent-offspring trios in the Deciphering Developmental Disorders (DDD) study and compared them with CNVs detected from exon-resolution array comparative genomic hybridization in 5197 probands from the DDD study.

**Results:**

Integrating calls from multiple ES-based CNV algorithms using random forest machine learning generated a higher quality data set than using individual algorithms. Both ES- and array comparative genomic hybridization–based approaches had the same sensitivity of 89% and detected the same number of unique pathogenic CNVs not called by the other approach. Of DDD probands prescreened with low-resolution CMAs, 2.6% had a pathogenic CNV detected by higher-resolution assays. De novo CNVs were strongly enriched in known DD-associated genes and exhibited no bias in parental age or sex.

**Conclusion:**

ES-based CNV calling has higher sensitivity than low-resolution CMAs currently in clinical use and comparable sensitivity to exon-resolution CMA. With sufficient investment in bioinformatic analysis, exome-based CNV detection could replace low-resolution CMA for detecting pathogenic CNVs.

## Introduction

Copy-number variants (CNVs) are differences in the number of copies of genomic segments and constitute a class of genetic variation of both medical and evolutionary importance. It has been estimated that 3% to 14% of patients with rare developmental disorders (DDs) harbor a pathogenic CNV.^1^, ^2^, ^3^ This diagnostic yield varies depending on the assay used for detection, the patient cohort, and clinical history of the patients.^4^^,^^5^ Most pathogenic CNVs have a dominant mode of inheritance and many arise de novo in patients with DDs. Population surveys have shown that the mutational mechanisms generating CNVs result in a continuous spectrum of CNV sizes, with smaller CNVs outnumbering larger CNVs^6^; however, among pathogenic CNVs, large CNVs predominate. This contrast is partly because of larger CNVs being more likely to be pathogenic and partly because of the low sensitivity of detecting smaller CNVs in clinical testing.

In most clinical genetics services, the primary assay used to identify CNVs in patients with DDs is chromosomal microarray (CMA) analysis. A range of different CMA assays are in widespread use, which vary in the size of CNVs that can be detected because of differences in the number, placement, and performance of the individual oligonucleotide probes on a given array. Many of these CMA assays use a genome-wide backbone of probes (often 60,000-180,000 probes) with the aim of detecting CNVs that are hundreds of kilobases in size or larger—here termed “low-resolution” CMA. Some CMA assays augment this genomic backbone with probes targeting exons of known dosage-sensitive genes to increase sensitivity to detect small pathogenic CNVs; however, CMA assays that target all protein-coding exons are not in widespread use.

As more clinical genetics services adopt next-generation sequencing for diagnosing DDs, it would be both advantageous and cost-effective to determine all classes of pathogenic genetic variation from this single assay. Both exome sequencing (ES) and genome sequencing interrogate all protein-coding exons and therefore, in principle, can detect the complete size range of CNVs affecting these exons. However, compared with small insertions/deletions (InDels) and single-nucleotide variants (SNVs), CNVs and structural variation, in general, remain more difficult to detect with high accuracy.^6^^,^^7^ A number of approaches have been developed,^8^ but, to our knowledge, there has been only 1 comparing the specificity and sensitivity of exon-resolution CMA versus ES-based approaches in very large patient cohorts.^9^

Here, we compare CNV detection from ES and genome-wide exon-resolution CMA in the DDD study cohort of 13,462 probands with severe DD recruited from the United Kingdom and Republic of Ireland, of which 9859 probands were sequenced as parent-offspring trios.^10^ Additionally, 5197 probands have exon-resolution CMA data available from a custom array comprising 1.11M probes targeting 97% of protein-coding exons of GENCODE transcripts (v2010-07-22)^11^ with multiple probes per protein-coding exon and an additional 0.67M genomic backbone probes in noncoding regions to give an overall median probe spacing of 2 kb (Supplemental Section Design of the DDD array-CGH microarray). Most (73%) but not all of the 9859 DD probands were prescreened by low-resolution CMA before recruitment to the DDD study, allowing us to assess the added diagnostic yield of exon-level resolution in CNV detection. We also present a systematic characterization of 598 de novo CNVs detected in the trios.

## Materials and Methods

### Sample and data collection

Children with severe, undiagnosed DDs were recruited for the DDD study by 24 centers across the United Kingdom and Ireland as previously described.^12^ The study performed genome-wide array comparative genomic hybridization (aCGH) on blood-derived DNA and ES of saliva- or blood-derived DNA. Among the 13,462 probands with ES data, 9859 were sequenced as parent-offspring trios, and, of these, 5197 had both proband exon-resolution aCGH and parent-offspring trio ES data available. Before enrollment into DDD 7182 (73%) of the 9859 participants were prescreened using clinical-grade CMA (typically a low-resolution array) to exclude patients with large pathogenic CNVs or aneuploidy.

### CNV calling from aCGH data

The custom CGH microarray (Agilent Microarray Design IDs: 031220/031221) used for CNV discovery was designed to have good power to detect single-exon CNVs by using 5 probes per exon, as well as having a dense backbone of approximately 320,000 intronic and intergenic probes with a median probe spacing of 2 kb. It is composed of 2 1 million probe Agilent arrays and has been designed to target genes and ultraconserved elements throughout the human genome. The aCGH CNV calls were obtained using a custom algorithm, CNsolidate (Supplemental Section S1).

### CNV calling from ES data

The ES data were generated using 1 of 2 custom bait designs based on the Agilent SureSelect v3 and v5 exomes, supplemented with additional probes targeting approximately 6000 high-value noncoding regions comprising 12.9% of the total targeted sequence.^13^ The sequencing was performed as described previously.^1^ The paired-end 75 bp sequencing reads were aligned onto the GRCh37d5 reference genome with bwa.^14^ Four programs were used to generate the initial raw callset: CANOES,^15^ CLAMMS,^16^ CoNVex,^17^ and XHMM^18^; the calls were then integrated using a random forest machine learning approach (Supplemental Sections S2-S4), their breakpoints resolved (Supplemental Sections S2 and S21), their inheritance status determined from overlapping parental calls, and their parent of origin ascertained from informative SNVs (Supplemental Section S18).

### Gene enrichment testing

To test enrichment in genes associated with DDs, we compiled a subset of 800 genes in which de novo variants are robustly associated with disease (ie, monoallelic and X-linked) from the Developmental Disorders Genotype-To-Phenotype Database (DDG2P,^19^ https://www.ebi.ac.uk/gene2phenotype/downloads/DDG2P.csv.gz version 2022-10-17) restricted to categories “definitive” or “strong.” In further text, we refer to these genes in which the de novo variant is sufficient to cause DDs simply as the “DN-DD genes.” For details, see Supplemental Section S6.

### Clinical evaluation of potentially pathogenic CNVs

CNVs were selected for clinical review by a DDD clinical reporting pipeline before this work^12^ and independently of the random forest classification described above. In the clinical reporting pipeline, all CNVs discovered by CNsolidate and CoNVex were filtered based on annotations provided by the 2 callers, CNV size, overlap with common and recurrent calls, and overlap with DDG2P genes (Supplemental Section S5). A set of 276 clinically validated pathogenic de novo CNVs from samples with both exon-resolution CMA and ES data available was used as a truth set to assess sensitivity of the methods.

### Overlap between ES and CMA calls

The final set of 598 de novo rare CNVs (Results and Supplemental Excel Chart) was annotated with the source caller, requiring 25% of the consensus CNV (across callers) to be overlapped by the original caller-specific call.

### Code availability

All code is freely available (Supplemental Section S3).

## Results

### Modeling-anticipated sensitivity of different CNV detection assays

Previous studies have suggested that CNV detection assays typically require at least 2 or 3 probes (or baits for ES) within the CNV for accurate detection. Therefore, to establish approximate baseline expectations of the sensitivity of different CNV detection assays to detect CNVs of different sizes (drawn from a population-based CNV size distribution established by the 1000 Genomes Project, see Supplemental Section S7), we performed simulations of the sensitivity of 4 different CNV detection assays: low-resolution CMA with either 60K (CytoSure Constitutional v3) or 180K (Agilent CGH ISCA v2) probes, the exon-resolution 2M CMA designed specifically for the DDD study, and ES (Supplemental Section S7). The custom exon-resolution CMA is expected to have the highest sensitivity because it targets most protein-coding exons with at least 5 probes. These simulations suggested that if we assumed that 2 probes/baits are sufficient for reliable CNV detection, then exon-resolution CMA would be expected to have 98% sensitivity for single-exon CNVs, whereas ES (which uses a single bait for most exons) would have 31% sensitivity, and the 180K and 60K CMA platforms would have 39% and 17% sensitivity, respectively. However, for CNVs that affect more than 3 exons, both exon-resolution CMA and ES-based CNV ascertainment would be expected to have 99% sensitivity, which exceeds by a wide margin the anticipated sensitivity of low-resolution CMA (39% and 69%; Supplemental Figure S8A). We note that the 180k CMA design that we modeled includes probes targeting exons of known dosage-sensitive genes^20^ and would be expected to detect 89% of simulated CNVs impacting DD-associated genes, compared with 60% by the 60k array and 99.9% by the 2M CMA and ES (Supplemental Figure S8B).

### CNV ascertainment in probands with severe DDs

Four ES-based CNV calling algorithms (CANOES,^15^ CLAMMS,^16^ CoNVex,^17^ and XHMM^18^) were run on 32,523 DDD proband and parental samples (Methods; Supplemental Section S2). To ascertain CNVs from the 5197 probands with exon-resolution CMA data, we used the CNsolidate algorithm (Supplemental Section S1). We observed differences in the number of calls between the 4 ES-CNV callers despite the same underlying data (Supplemental Figure S9), ranging from an arithmetic mean of 15 calls per sample from XHMM to 67 calls per sample from CoNVex. The initial union ES-based CNV callset consisted of 9.6M calls with unique breakpoints within a sample but included possible duplicate calls with discrepant breakpoints produced by different programs. After merging potentially redundant calls (Supplemental Section S2), 7.3M nonredundant CNV calls remained (Supplemental Figure S10A).

To integrate the 4 ES-based CNV callers and generate a combined callset of improved accuracy, we annotated each ES-based CNV call with a range of quality-related metrics and trained a random forest classifier on 1332 common CNVs (Supplemental Section S2). We trained separate random forest models for deletions and copy-number gains (in further text referred to as “duplications”); the most informative variables differed between the 2 models (Supplemental Figure S23). In total, 54,607 calls (0.75%) passed the random forest filtering threshold (Supplemental Section S11) of which 718 (1.3%) were not observed in parents of the trios (ie, were putative de novo CNVs). We further excluded 120 (17%) of these putative de novo CNVs because 15 (2%) were in regions of the genome that are known to rearrange in blood cell lineages and 82 (11%) were also observed at implausibly high frequencies (*N* > 24) in 17,208 unrelated, unaffected individuals (parents of other trios) and were thus unlikely to be pathogenic (the threshold of *N* = 24 was set based on prevalence of known pathogenic recurrent CNV syndromes in the same set of individuals^21^). A further 23 CNVs (3%) were observed in healthy control samples in public databases of structural variation (*N* > 24 in the Database of Genomic Variants^22^ or allele frequency > 0.01 in 1000 Genomes Project^23^ or GnomAD-SV^6^) and are thus also unlikely to be pathogenic. The final ES-based callset used in subsequent analyses thus consisted of 598 de novo rare CNVs in 9859 probands (Supplemental Figure 10B).

**Figure 1:**
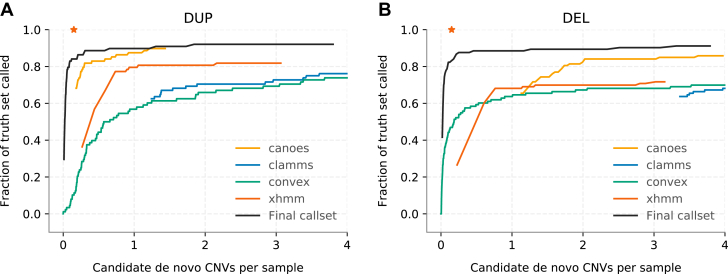
**Pseudo-ROC curves for duplications (A) and deletions (B) show sensitivity of individual ES callers and our final callset versus the number of de novo calls per sample under varying quality cutoffs.** The truth set consists of 276 clinically validated pathogenic copy-number variants (CNVs) discovered mainly from the exon-resolution chromosomal microarray. The red asterisk in each plot denotes 0.15 putative de novo CNVs called per sample. DEL, deletions; DUP, duplications; ES, exome sequencing; ROC, receiver operating characteristic.

Comparison of the individual and combined ES-based CNV callsets to a truth set of 276 clinically validated pathogenic CNVs largely discovered from the DDD exon-resolution CMA showed that the accuracy of all individual ES-CNV callers was considerably lower than that of the combined random forest callset (Figure 1). For example, if controlling false-positive calls equally by restricting each callset to a maximum of 0.15 putative de novo CNVs called per sample,^4^^,^^5^ the sensitivity to de novo CNVs of individual ES callers would be between 10% to 68% for duplications and 26% to 65% for deletions. In comparison, the predicted sensitivity of the integrated random forest callset at this stringent filtering threshold was 84% for both duplications and deletions.

We next used the set of 276 clinically validated CNVs to compare sensitivity of the integrated ES-based approach with exon-level CMA. To perform this comparison at the same level of specificity in both platforms, we first created a CMA-based callset using the recommended CNsolidate threshold of 0.5 and used it to determine filtering thresholds to create an ES-based callset (0.37 for duplications and 0.45 for deletions). We then assessed the sensitivity using 2 metrics: (1) a lenient approach whereby any overlap (≥1 bp) between a clinically validated CNV and an ES-based CNV call was sufficient and (2) a stricter approach in which we required 25% reciprocal overlap. We found that 261 (95%) of the clinically validated CNVs overlapped an ES-based call by ≥1 bp and 240 (87%) when a 25% reciprocal overlap was required (Supplemental Figure S12). We note that the difference in sensitivity between the 2 metrics was driven by single-exon events in which cross-platform differences in probe placement and CNV breakpoint estimation have a greater effect on call overlaps. For large CNVs (ie, >10 exome baits), ES-based ascertainment was at least as sensitive as exon-resolution CMA; filtered ES-based calls achieved 98% sensitivity, whereas the sensitivity of exon-resolution CMA calls was only 92% at the applied thresholds. In general, most of the pathogenic CNVs missed by ES (18/30, 60%) intersected either 1 (9/30) or 0 (9/30) exome baits and are thus inaccessible to discovery using ES-based methods (Supplemental Figure S12). Although most pathogenic CNVs missed by exon-resolution CMA or ES-based calling had small (<10) numbers of probes/baits, a few larger CNVs (4/30 of those missed by ES-based calling) were missed because of being fragmented into smaller number of calls, none of which met the required quality thresholds. In comparison, the exon-resolution CMA identified 246 of the 276 clinically validated CNVs (89%) with overlap of ≥1 bp and 245 (89%) when a 25% reciprocal overlap was required (Supplemental Figure S12).

Among the 598 rare de novo CNVs, we identified 182 that had copy-number data available from both blood and saliva, typically with each tissue profiled on a different platform. Of these, 37 were discordant between the 2 platforms; 5 had log2 ratios in the ES data consistent with being a mosaic event (>−0.621 for deletions or <0.379 for duplications). These analyses suggest that discrepancies between the CNV calling on the 2 platforms are bigger contributors to these discrepancies than differential mosaicism between tissues. This finding is supported by previous analyses where mosaic variants were detected in 0.22% to 0.34% of the patients from the DDD study.^24^^,^^25^

### Characteristics of de novo CNVs

**Figure 2:**
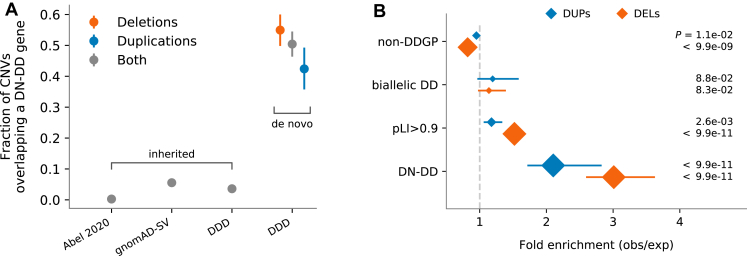
**De novo CNVs overlapping DN-DD genes.** A. Fraction of de novo copy-number variants (CNVs) overlapping a DN-DD gene compared with inherited CNVs in the Deciphering Developmental Disorders (DDD) and 2 other large-scale studies. B. Enrichment of de novo CNVs in genes in which a de novo variant is sufficient to cause a disease (DN-DD), in constrained genes with high pLI score (pLI > 0.9), in recessive biallelic genes (biallelic DD), and in genes not previously associated with developmental disease (non-DDGP). The intervals show 90% of the simulated distribution (Supplemental Figure S14), and the size of the diamonds indicates the significance of the results with *P* values shown on the right. DEL, deletions; DUP, duplications.

Among the 598 high-quality de novo CNVs we detected, de novo deletions were more frequent than duplications (64% vs 36%), consistent with other large-scale studies.^6^^,^^26^ Sixty-five percent (*N* = 391) of de novo CNVs affected the coding sequence of multiple genes, whereas 32% (*N* = 194) affected the coding sequence of a single gene (Supplemental Figure S13), and 3% (*N* = 13) did not affect coding sequence. Among single-gene CNVs, only 10% overlapped all coding exons of the gene; most were partial-gene CNVs. Approximately half of de novo CNVs intersected DD-associated genes in which de novo variants can be sufficient to cause disease (DN-DD genes) (Figure 2A). This proportion is much higher than the proportion of exome baits that target exons of DN-DD genes (6.6%) and is much higher than the proportion of inherited CNVs that encompass DN-DD genes, either in population studies or in the DDD families. A permutation test (Supplemental Section S6), assuming a uniform genome-wide CNV mutation rate, confirmed that DDD probands have significantly more de novo deletions and duplications that affect DN-DD genes (2.0× for duplications and 3.0× for deletions; *P* < 1e-10) than expected by chance. The same test also showed that de novo deletions and duplications are significantly enriched in a broader set of constrained genes (1.2× and 1.5×; *P* = 2.6e-3 and *P* < 1e-10) with high probability of intolerance to heterozygous loss-of-function variants^27^ (pLI > 0.9) (Supplemental Figure S14) but not in biallelic DD-associated genes or in genes that are not DD-associated (Figure 2B).

The enrichment of de novo duplications in DD-associated genes could be driven by triplosensitivity or by gene-disrupting duplications. Among duplications affecting a single gene, we observed that a higher proportion of partial-gene duplications (28%, 13/46) than entire gene duplications (9%, 1/11) affects DN-DD genes (Supplemental Figure S15). This is in stark contrast to deletions affecting a single gene, where we observed a lower proportion of partial-gene deletions (51%, 66/129) than entire gene deletions affecting DN-DD genes (88%, 7/8). This suggests that an appreciable proportion of pathogenic de novo duplications are likely to be gene disrupting rather than operating via triplosensitivity.

**Figure 3:**
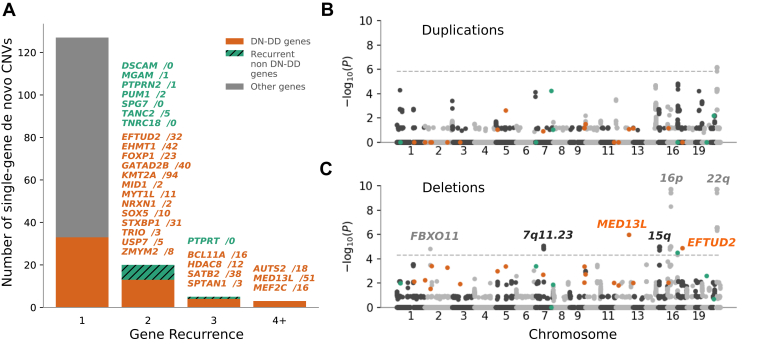
**Gene recurrence in de novo CNVs.** A. Gene recurrence in the 195 de novo copy-number variants (CNVs) that overlapped the coding sequence of a single gene. The gene names are followed by the number of samples with a protein truncating variant in patients with developmental disorders (DD) from a recent analysis of 31,058 trios^28^; most of the genes that were affected multiple times across the patients from the Deciphering Developmental Disorders study were either known or novel candidate DD genes in the study. B and C. Gene recurrence in de novo CNVs overlapping coding sequence of any number of genes separately for duplications (B) and deletions (C). Highlighted in gray/black text are known neurodevelopmental loci, which harbor statistically significant genes according to our test (Supplemental Figure S15). *P* values were determined by a permutation test, which consisted of 5e9 iterations each under an assumption of a uniform CNV rate. The dashed line marks a false discovery rate of 0.01 as calculated by the Benjamini-Hochberg procedure.

To investigate genes specifically associated with DDs caused by CNVs, we first looked at single-gene CNVs. Twenty-eight genes were affected recurrently by the 195 de novo single-gene CNVs, of which 20 were known DN-DD genes (Figure 3A^28^). Of the remaining 8 genes, 3 (*PUM1*, *SPG7*, and *TANC2*) are associated with other neurological monogenic disorders in OMIM (www.omim.org) and 1 (*PTPRT*) already has partial but inconclusive evidence of association with neurodevelopmental disorders.^29^^,^^30^

We then tested for gene-specific enrichment of CNVs across the complete set of 598 de novo CNVs, including those affecting multiple genes, using a genome-wide permutation test assuming a uniform CNV rate (Methods). In total, 168 genes passed genome-wide significance threshold (Benjamini-Hochberg–corrected *P* < .01; Figure 3B-C^28^); 10 genes (Supplemental Table S16A) were known DN-DD genes.^19^ Of the remaining 158 genes that passed genome-wide significance, all but 1 are located within or flanking (±1 Mbp) known DD-associated recurrent pathogenic CNVs (Supplemental Table S16B).^31^ The remaining significant gene, *SPG7*, has previously been associated with autosomal recessive spastic paraplegia.^32^ To assess if *SPG7* is a potential DN-DD gene candidate, we performed in-depth clinical review of all 3 patients with deletions intersecting *SPG7*. Two of 3 de novo *SPG7* deletions also overlap either the coding sequence or promoter of the flanking well-characterized DN-DD gene *ANKRD11*, and both patients presented with phenotypes consistent with *ANKRD11* loss, whereas the third patient also has a de novo protein-truncating SNV in the gene *KMT2A* consistent with their symptoms. As such, we consider *SPG7* to be a likely passenger alongside the pathogenic partial deletions of *ANKRD11*.

### Association of de novo CNVs with parental age and sex

**Figure 4:**
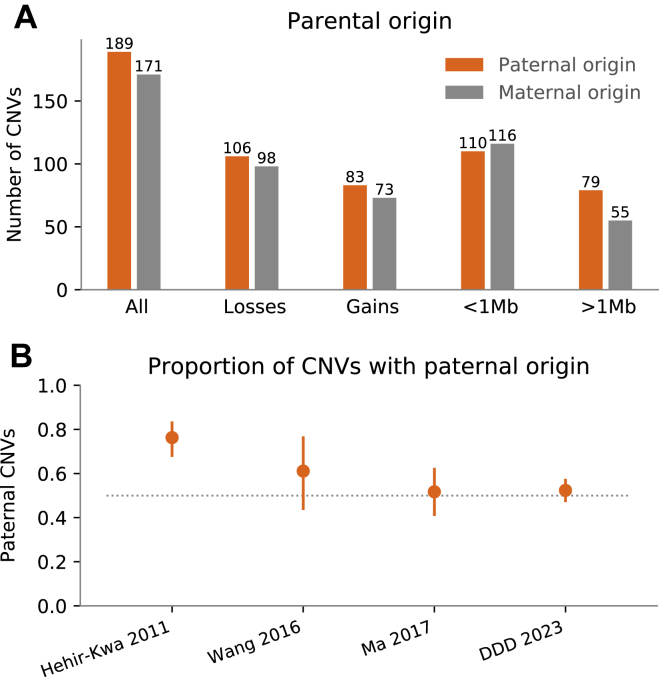
**Parental origin of de novo CNVs.** A. De novo copy-number variants (CNVs) identified as part of this study quantified by paternal and maternal origin. None of the categories are significantly enriched for paternal CNVs (Benjamini-Hochberg–corrected 2-sided binomial tests, *P* > .05). Although we do not find a statistically significant difference between de novo CNVs of paternal or maternal origin (*P* = .37), we observe a higher absolute number of de novo CNVs of paternal origin in our data; however, it is much less prominent than expected from Hehir-Kwa et al.^35^ B. Proportion of de novo CNVs with paternal origin in 4 studies. DDD, Deciphering Developmental Disorders.

We next sought to determine if there was any parental bias in the origin of de novo CNVs (Supplemental Sections S17 and S18). A strong paternal bias has been observed for other classes of variation (eg, approximately 80% for SNVs^33^ and approximately 75% for InDels^34^), but the evidence for CNVs has been mixed; previous studies have observed strong, weak, or absent paternal bias^35^, ^36^, ^37^ but also a strong maternal bias at specific loci or for aneuploidies.^38^^,^^39^ We were able to determine parental origin for 360 (64%) of de novo autosomal CNVs, 189 (53%) of which had paternal origin, a nonsignificant bias (binomial test *P* = .40; Figure 4,^35^ Supplemental Table S19). There were no obvious parental biases when stratifying these CNVs into deletions and duplications or larger and smaller events (Figure 4A^35^).

Similarly, the mutation rate of de novo SNVs is known to increase markedly with paternal age^33^ and more modestly with maternal age.^28^ Moreover, the risk of aneuploidies is known to increase with maternal age,^40^ but an association between parental age and increased de novo CNV has not been established.^41^ We did not observe a significant association between de novo CNVs and parental age (Supplemental Figure S20). Although we can be confident that any parental age effect for CNVs must be significantly smaller than for SNVs (*P* < 1e-270), larger studies are required to determine whether a much more modest parental age effect might exist.

### Pathogenic de novo CNVs in DDD ascertained from ES

Of the 598 de novo CNVs identified via ES-based calling, we identified 305 (51%) as plausibly pathogenic either because of encompassing many genes as per American College of Medical Genetics and Genomics and ClinGen guidelines^42^ or because of affecting a known DD-associated gene based on DDG2P^18^ (Supplemental Figure S10B). A clinical review of these patients confirmed that these variants were likely to be contributing to the proband’s disorder (Supplemental Excel Chart), a diagnostic yield of around 3.1%. We note that 86 of 305 (28%) of these pathogenic CNVs overlapped fewer than 3 probes in low-resolution 60k CMA and would likely be missed by this assay.

We also examined whether any of the de novo CNVs might be contributing to recessive disorders by seeking gene-disrupting variants on the other allele. We identified 7 instances of additional truncating variants affecting the same gene as the de novo CNV; however, all were common in the general population (allele frequency > 0.2) and are thus unlikely to be pathogenic.

With the inclusion of thousands of ultraconserved noncoding elements in our custom ES,^13^ we also sought noncoding de novo CNVs plausibly associated with patient phenotype. In addition to the pathogenic noncoding deletion affecting the 5′ untranslated region of *ANKRD11* described above, we found 3 additional patients with a phenotype fully explained by a noncoding deletion: 2 intersected the promoter of *MEF2C* and are described in more detail elsewhere,^43^ and 1 intersected the promoter of *MBD5*. We also identified an additional patient with a deletion within the promoter of *EHMT1* for which there is currently insufficient evidence to classify as being pathogenic or likely pathogenic.

**Figure 5:**
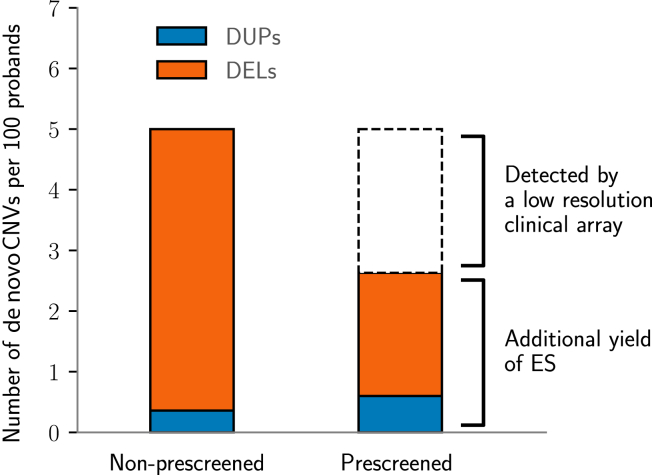
**Different discovery rates of pathogenic de novo copy-number variants (CNVs) were observed in the groups of chromosomal microarray (CMA)-untested (14 of 280; left) and CMA-tested (189 of 7182; right) patients.** DEL, deletions; DUP, duplications; ES, exome sequencing.

Before recruitment to the DDD study, 7182 of the 9859 participants (73%) with trio ES data had previously been clinically tested for large pathogenic CNVs using low-resolution CMA. As such, DDD study participants do not represent an unbiased sample of patients with DDs but rather will be depleted of patients with large pathogenic CNVs. Among those who had undergone prior low-resolution CMA testing, we observed 2.6% with a pathogenic CNV identified by 1 or both of the exon-resolution CMA and ES-based CNV detection. Among the 27% of participants who had not previously received low-resolution CMA, we identified 280 participants (3%) who were likely recruited before CMA testing was available in their regional center. In this group, we observed a higher diagnostic yield from de novo CNVs of 5.0%. Comparison of the CNV diagnostic yields in these 2 groups suggests that 52% of pathogenic CNVs detectable from ES are invisible to low-resolution arrays (rate ratio test, CI = 31% to 98%, *P* = .044; Figure 5), which is consistent with our simulations (Supplemental Figure S8). The CNV diagnostic yield of CMA testing in larger cohorts of patients has been shown in previous studies^4^^,^^5^ to be higher, in the range of 10% to 15%. Therefore, our estimate of the added value of CNV detection from ES of 52% is likely to vary depending on the ascertainment of the cohort under study.

## Discussion

We developed a CNV calling workflow from ES data that integrates 4 different calling algorithms using random forest machine learning to generate an ES-based CNV callset of considerably higher quality than achievable with any single calling algorithm. Applying this CNV calling workflow to 9859 parent-offspring trios participating in the DDD study identified pathogenic CNVs that could not be detected by low-resolution CMA, often small CNVs encompassing few exons. Modeling of sensitivity based on probe/bait locations and empirical detection of pathogenic CNVs in a subcohort that had not received prior CMA screening suggests that this ES-based CNV calling workflow likely has high sensitivity to detect the typically large pathogenic CNVs that can be detected by low-resolution CMA.

ES-based CNV calling has previously been compared with, and found to have lower sensitivity than, aCGH-based approaches^9^; however, in this published comparison, a single algorithm was used that used a simple *z*-test, before more recent technological developments in ES-based CNV calling. Low precision and variable performance of any single ES-based CNV caller was recently shown in a benchmarking exercise focused on a gold standard data set^44^ and in a study of 503 patients that demonstrated the value of using machine learning to integrate ES-based CNV calls using 4 CNV callers partially overlapping those used here.^45^ These previous results, together with our findings, demonstrate the necessity of combining calls from multiple algorithms with robust filtering approaches. We note that more recent methods were published while this work was under review, which also supports our finding that multi-exon CNVs from ES data can have high concordance with CNVs called from other platforms.^46^ We also note that our random forest model required several iterations of manual curation to optimize and that the optimal parameters of the random forest model depend on the noise properties of the underlying data and may need to be retrained for different data sets. The ES data analyzed in this study was generated on a highly standardized high-throughput pipeline and therefore may have more consistent noise properties than smaller, more heterogeneous data sets.

Comparison of ES-based CNV calling with exon-resolution CMA in 5197 families suggested that the 2 approaches have similar sensitivity for pathogenic CNVs. Each approach has incomplete sensitivity to detect CNVs that encompass 1-3 exons, with the result that the combination of the 2 approaches, although largely concordant, did identify more pathogenic CNVs that would be detected by either approach in isolation. Of DDD probands prescreened with low-resolution CMA, 2.6% had a pathogenic CNV detected by higher-resolution assays, and the diagnostic yield of pathogenic CNVs in patients that have not previously been screened with low-resolution CMA was 5.0%. This is much lower than the >10% diagnostic yield that has been reported in similar patient cohorts, but the difference of 2.4% is comparable to the 1.3% added diagnostic yield reported in a previous, smaller study of patients with neurodevelopmental disorders, the majority of whom had previously been screened with low-resolution CMA.^47^ We did note that a low proportion of large pathogenic CNVs were hard to call from both exon-resolution assays because of fragmentation into smaller CNV calls by the calling algorithms. This suggests that there are additional improvements to be made in the bioinformatic post-calling merging of CNV calls to support robust clinical interpretation with accurate breakpoint definitions with respect to the genes affected by a CNV.

Overall, we identified 598 de novo CNVs in the 9859 parent-offspring trios, of which 305 were clinically interpreted to be contributing to the proband’s clinical phenotype (ie, classified as pathogenic or likely pathogenic). We did not observe an age or sex bias in the parental origin of these de novo CNVs. The lack of sex bias is in contrast to previous, smaller studies that have suggested a paternal bias for de novo CNVs.^35^^,^^36^ This discordance may be due, in part, to the different size distributions and associated mutational mechanisms being interrogated in the previous studies. Meta-analyzing our current data with 3 previous studies (Supplemental Table S19) does suggest that there may be a relatively subtle paternal bias (58%:42%), but larger data sets across the full size range of pathogenic CNVs would help to confirm this.

Population surveys of CNVs across the full-size distribution have repeatedly shown that smaller CNVs, below the threshold of detection of low-resolution CMA, are far more numerous and generated at higher mutation rates than larger CNVs. Nonetheless, this study, in combination with previous work, clearly shows that the added diagnostic yield from detecting these smaller CNVs is relatively modest. What matters more in a clinical context is not the total number of CNVs of a given size class but rather the size distribution of CNVs that disrupt developmentally important genes, which is clearly biased toward very large CNVs that can be detected by low-resolution CMA. In the context of large-scale diagnostic testing of tens of thousands of patients with DDs, ES-based CNV calling is likely to enable a diagnosis in hundreds of families who might well otherwise go undiagnosed. One limitation of our study is that we cannot estimate directly the overall diagnostic yield of ES-based CNV calling as a first-line test due to the prior clinical CMA testing for most of the DDD cohort.

One of the limitations of ES-based CNV calling, as opposed to the custom exon-resolution CMA assay that we used, is the lack of baits to noncoding sequences, meaning that ES-based calling has lower precision in determining the breakpoints of a CNV. This limits the potential for ES-based CNV calling to detect pathogenic CNVs affecting noncoding regulatory elements. In theory, this limitation could be overcome by including a genome-wide “backbone” of noncoding baits in a customized exome design; however, we doubt that, currently, the added diagnostic yield from greater breakpoint precision will be worth the added sequencing costs incurred. Given the high sensitivity of ES-based CNV calling to large pathogenic CNVs detectable by low-resolution CMA, such genome-wide backbone baits are not necessary for ES-based CNV calling to detect these large pathogenic CNVs in the absence of low-resolution CMA. Customizing exome designs to include additional baits flanking exons of dosage-sensitive genes to improve sensitivity to detect single-exon deletions might be a preferable approach to increase sensitivity of ES-based CNV calling. In principle, increasing the depth of coverage of standard ES should also increase sensitivity for calling single-exon CNVs (and for detecting mosaic CNVs).

Our study provides compelling evidence from side-by-side comparison in thousands of families of exon-resolution CMA and ES-based CNV calling that, with appropriate development and deployment of a bioinformatic workflow integrating multiple calling algorithms, ES-based CNV calling has higher sensitivity for pathogenic CNVs than low-resolution CMA and can even render exon-resolution CMA largely redundant. We look forward to similarly scaled side-by-side comparisons of other genomic assays that purport to increase diagnostic yield of pathogenic structural variants (eg, short or long read genome sequencing technologies) to accurately quantify the added diagnostic yield over and above the application of best practice bioinformatics pipelines to cheaper assays, enabling diagnostic services to make well-informed cost/benefit decisions.

## Data Availability

Sequence and variant-level data and phenotypic data for the DDD study data are available from the European Genome-phenome Archive (EGA; https://www.ebi.ac.uk/ega/) with study ID EGAS00001000775. Clinically interpreted variants and associated phenotypes from the DDD study are available through DECIPHER (https://www.deciphergenomics.org).

## ORCIDs

Petr Danecek: http://orcid.org/0000-0002-4159-1666

## Conflict of Interest

Matthew E. Hurles is a co-founder of, consultant to, and holds shares in, Congenica Ltd, a genetics diagnostic company. Eugene J. Gardner is an employee of and holds shares in Insmed, Inc. All other authors declare no conflicts of interest.
